# Influence of storage temperature and time on the physicochemical and bioactive properties of roselle-fruit juice blends in plastic bottle

**DOI:** 10.1002/fsn3.97

**Published:** 2014-02-27

**Authors:** Beatrice Mgaya-Kilima, Siv Fagertun Remberg, Bernard Elias Chove, Trude Wicklund

**Affiliations:** 1 Department of Chemistry, Biotechnology and Food Science, Norwegian University of Life SciencesP.O. Box 5003, 1432, Åas, Norway; 2 Department of Food Science and Technology, Faculty of Agriculture, Sokoine University of AgricultureP.O. Box 3006, Morogoro, Tanzania; 3 Department of Plant and Environmental Sciences, Norwegian University of Life SciencesP.O. Box 5003, 1432, Åas, Norway

**Keywords:** Anthocyanins, antioxidants, fruit juices, roselle

## Abstract

Roselle-fruit juice blends were made from roselle extract and mango, papaya, and guava juices at the ratio of 80:20, 60:40, 40:60, and 20:80, % roselle: fruit juice, respectively. The blends were pasteurized at 82.5°C for 20 min and stored in 100 mL plastic bottles at 28 and 4°C for 6 months. The effects of storage time and temperature on physicochemical and bioactive properties were evaluated. Total soluble solids, pH, and reducing sugars increased significantly (*P* < 0.05) in some blends while titratable acidity decrease with increasing storage time. Vitamin C, total monomeric anthocyanins (TMA), total phenols (TPC), and antioxidant activity (ferric reducing ability of plasma, FRAP) in all roselle-fruit blends (40% roselle) decreased significantly (*P* < 0.05) at 28 and 4°C as storage progressed. Vitamin C in all roselle-fruit blends (40% roselle) decreased from 58–55% to 43–42% when stored at 28 and 4°C, respectively. TMA losses were 86–65% at 28°C and 75–53% at 4°C while TPC losses were 66–58% at 28°C and 51–22% at 4°C. Loss of antioxidant capacity (FRAP) was 18–46% at 28°C and 17–35% at 4°C. A principal component analysis (PCA) differentiated roselle-juice fruit blends into two clusters with two principle components PC1 and PC2, which explained 97 and 3% (blends stored at ambient temperature) and 96 and 4% (blends stored at refrigerated temperature) of the variation, respectively. PC1 differentiated roselle-guava juice blends which were characterized by vitamin C, TPC, FRAP, and pH, while PC2 from another cluster of roselle-mango and roselle-papaya juice blends and was characterized by TSS, RS, and color parameters (L* a* b*). However, TMA was the main variable with the highest effect on all roselle-fruit juice blends regardless of the storage time and temperature.

## Introduction

*Hibiscus sabdariffa* L. (family *Malvaceae*), commonly known as roselle, red sorrel, or karkadè, is widely grown in Africa, South-East Asia, and some tropical countries of America (Abou-Arab et al. 2001; Sagayo-Ayerdi et al. 2007; Amor and Allaf 2009; Cisse et al. 2011). Roselle produces red edible calyces with a unique flavor and brilliant red color. The calyces are commonly used to make jelly, juice, jam, wine, syrup, pudding, cake, ice cream, and flavour (Tsai et al. 2002; Tsai and Huang 2004; Duangmal et al. 2008). The beverages produced by roselle calyce*s* are called hibiscus tea, bissap, roselle, red sorrel, agua de Jamaica, Lo-Shen, Sudan tea, or karkadè (McKay et al. 2010).

The calyx contains two main anthocyanins, delphinidin-3-sambubioside, also known as delphinidin-3-xylosylglucoside or hibiscin, and cyanidin-3-sambubioside, also known as cyanidin-3-xylosylglucoside or gossypicyanin, and two minor anthocyanins, delphinidin-3-glucoside and cyanidin-3-glucoside (Wong et al. 2002; Amor and Allaf 2009; Cisse et al. 2011). Roselle anthocyanins can contribute to health benefits as a good source of antioxidants as well as a natural food color (Tsai et al. 2002; Duangmal et al. 2008). They are derivatives of the basic flavylium cation structure with an electron-deficient nucleus, which makes them highly reactive and their reaction involves discolorization of the anthocyanin pigments (Chumsri et al. 2008). Factors like light, pH, temperature, oxygen, ascorbic acid, and sugar are contributing factors in degradation or stability of anthocyanins (Fennema 1996; Tsai and Huang 2004; Chumsri et al. 2008; Cisse et al. 2011).

Most people do not prefer beverages made from roselle extract as it has an acidic and bitter taste. Blending roselle extract with tropical juice from fruits such as mango, guava and papaya could improve the aroma, taste, nutritional, and antioxidant properties of the roselle-fruit blends. The fruits chosen in this study were due to the availability of these fruits during the season. Papaya and guava are also among the underutilized fruits in fruit juice production.

Guava (*Psidium guajava* L.) belongs to the family *Myrtaceae*, commonly known as the apple of the tropics. It grows well in tropical and subtropical regions. The fruits are rich in vitamin C and are almost fivefold higher when compared with oranges (Jawaheer et al. 2003; Ashaye et al. 2005; Thaipong et al. 2006) Most of the guava produced around the world is consumed fresh (Jawaheer et al. 2003).

Papaya (*Carica papaya* L.) is grown in every tropical and subtropical country. A tree-like herbaceous crop, it is a member of the Caricaceae family. It is one of the largest in size of the tropical fruits; it has a pulpy flesh yellow or orange colored with shades of yellow and red, depending on the fruit variety. It has the flavour of a cantaloupe; sweet and juicy with some muskiness (Parker et al. 2010). The fruits are very nutritious due to high contents of vitamin A, C, and iron (Chowdhury et al. 2008).

Mango (*Mangifera indica* L) is one of the most important and widely cultivated fruits of the tropical and subtropical world (Akhter et al. 2012). It is also known as the king of the tropical fruits (Gerbaud 2008). It is an excellent source of fiber, vitamins A, C, and B complex, iron, and phosphorus (Akhter et al. 2012).

Many studies have been conducted on physicochemical and antioxidant properties of roselle extract (Tsai and Huang 2004; Chumsri et al. 2008; Cisse et al. 2011). However, few studies have been conducted on roselle-fruit juice blends, and practically none on the effects of storage time and temperature on roselle-fruit juices. The aim of the present study was to investigate the influence of storage time and temperature on the physicochemical and antioxidant properties of roselle-fruit blends stored in plastic bottles.

## Materials and Methods

### Plant material

Dark red dried roselle calyces were purchased from the municipality market in Morogoro. Guava (pink), papaya (Solo), and mango (Dodo) were purchased from the horticulture garden at Sokoine University of Agriculture, Tanzania.

### Preparation of roselle extract

Dried roselle calyces (10% moisture content) were ground for 1 min using a blender (Kenwood BL 440, Kenwood, Boulogne, France). Roselle calyces were ground at a ratio of 1:10 (roselle:water) and extracted using a water bath at 50°C for 30 min as described previously (Chumsri et al. 2008), and filtered through a cheese cloth.

### Fruit juice preparation

Fully matured and high-quality fruits of mango, papaya, and guava were used. Fresh fruits were thoroughly washed, peeled, cut into small pieces (guava were not peeled), and put in a juice extractor (Kenwood JE 810, Edinburgh, U.K.).

### Preparation of roselle-fruit juice blends

Three beverage product categories of roselle-mango, roselle-papaya and roselle-guava were formulated in the ratio of 80:20, 60:40, 40:60, and 20:80 roselle extract: fruit juice, respectively. Sodium benzoate (1 g/L) and citric acid (1 g/L) were added to all roselle-fruit blends as preservatives.

The juices were filled in 100 mL plastic bottles, loosely capped, and pasteurized in a water bath at a temperature of 82.5°C for 20 min and cooled rapidly to room temperature by immersing the bottles in a cold water bath. Samples were drawn for initial chemical analyses and thereafter analyses were carried every month for 6 months.

### pH, titratable acidity and total soluble solids

pH, titratable acid (TA) and total soluble solids (TSS) of roselle-fruit blends were determined according to AOAC (1995). pH was measured using a Hanna portable pH meter (HANNA HI9125, Cluj-Napoca, Romania). TA was determined using 0.1 N sodium hydroxide and phenolphthalein as an indicator and was expressed as % malic acid, while TSS was measured with a hand refractometer (Mettler Toledo, Schwerzenbach, Switzerland) and expressed as Brix.

### Reducing sugars

Reducing sugars (RS) were determined by the Luff-Schoorl method as described by Egan et al. (1981). Two grams of sample was weighed in a 100-mL measuring flask and 90 mL hot distilled water, 5 mL Carrez I and 5 mL Carrez II solution were added. The solution was mixed and filtered using a Whatman filter (no. 542), and 10 mL of filtrate was transferred into a 250-mL Erlenmeyer flask, followed by adding 10 mL of copper reagent and swirled. The solution was then boiled in a direct flame for 3 min, cooled in a water bath followed by the addition of 1 g potassium iodide and 10 mL 6 N HCL. The mixture was then titrated with 0.1 N Na_2_S_2_O_3_ until a yellow color appeared, 1 mL of starch solution was added and the mixture was titrated continuously until a blue color appeared. RS was determined by interpolation in a table (Egan et al. 1981) after subtracting the blank assay to the volume of sodium thiosulfate of the titration. The results are expressed as mg/100 g fresh weight (FW).

### Vitamin C assay

Vitamin C content for the roselle fruit juices was determined according to the method of Dashman et al. 1996 with some modifications using Folin-Ciocalteu reagent (FCR). Twenty milliliters of sample was pipetted into a 100-mL volumetric flask followed by 2 mL of 10% tetrachloroacetic acid solution and diluted to 100 mL with distilled water. The sample was poured into a conical flask, swirled gently for 1 min and left to stand for 1 min and filtered with a Whatman filter (no. 542). One milliliter of sample or standard solution (3 mg ascorbic acid in 1 mL distilled water) was pipetted into a test tube followed by the addition of 3 mL distilled water and 0.4 mL of FCR and incubated at room temperature for 10 min. The absorbance was read at 760 nm using a Jenway 6405 UV–VIS spectrophotometer (Essex, U.K.). The results were expressed as mg/100 g FW.

### Determination of antioxidant activity

The antioxidant activity for the roselle fruit blends was determined by the ferric reducing ability of plasma (FRAP) assay (Benzie and Strain 1996) with some modifications. Three milliliters of freshly prepared FRAP solution (0.3 mol/L acetate buffer [pH 3.6] containing 10 mmol/L 2,4,6-tripyridyl-s-triazine [TPTZ] in 40 mmol HCl and 20 mmol/L FeCl_3_·6H_2_O) and 100 μL of sample or standard was incubated at 37°C for 4 min and the absorbance was measured at 593 nm using a spectrophotometer. An intense blue color is formed when the ferric-tripyridyl-s-triazine (Fe^3+^-TPTZ) complex is reduced to the ferrous (Fe^2+^) form. A range of iron sulfate concentrations from 0.25 to 2.0 mmol/L was used to prepare a calibration curve. The results are expressed as millimoles of Fe^2+^ per liter of FW (mmol Fe^2+^/L FW).

### Total phenolic assay

Total phenolic content (TPC) for the roselle fruit blends was determined according to the Folin-Ciocalteu method (Singleton et al. 1999) with modifications. An aliquot of 300 μL sample solution was mixed with 1.5 mL of Folin-Ciocalteu reagent (diluted 10 times), and 1.2 mL of sodium carbonate (7.5% w/v). After incubation at room temperature for 30 min in the dark, the absorbance was measured at 765 nm in using a spectrophotometer. Gallic acid (0–500 mg/100 g) was used for calibration of a standard curve. The results are expressed as milligrams of gallic acid equivalents per 100 g of FW (mg GAE/100 g FW).

### Total monomeric anthocyanin content

Total monomeric anthocyanin (TMA) content for roselle-fruit juice blends was determined using the pH differential method (Lee et al. 2005). The absorbance was measured at 520 and 700 nm using a spectrophotometer. The absorbance (*A*) of the sample was then calculated according to the following formula:


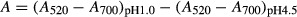


The monomeric anthocyanin pigment content in the original sample was calculated according to the following formula:


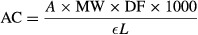


where *A* is the difference of sample absorbance between pH 1.0 and 4.5, *ε* is the molar extinction coefficient for cyanidin-3-glucoside (26,900 L/mol/cm), *L* is the path length of the spectrophotometer cell (1.0 cm), DL is the dilution factor and molecular weight (MW) of cyanidin-3-glucoside (449.2 g). The results are expressed as mg cyanidin-3-glucoside equivalent/100 g extract (mg/100 g) FW.

### Statistical analyses

Analysis of variance (ANOVA) was applied using a factorial design with two factors including storage temperature (28 and 4°C) and storage time (0, 1, 2, 3, 4, 5, and 6 months). The effect of each factor on the response variable (TSS, pH, TA, RS, vitamin C, FRAP, TMA, TPC) as well as the effects of interactions between the different factors were tested. Significance was accepted at *P* < 0.05 using Minitab Statistical Software (Version 16.0, 2008; Minitab Statistical Software, Minitab Inc., Enterprise Drive State College, PA). ANOVA was only performed on all roselle-fruit juice blends with 40% roselle (40R) as all blends showed a similar trend. Principal component analysis (PCA) was applied to analyze the relationship between roselle-fruit blends (80, 60, 40, 20% roselle) and storage time (0, 1, 2, 3, 4, 5, 6 months) and temperature (ambient and refrigerated) using Unscrambler X 10.2 (Camo Process AS, Oslo, Norway).

## Results and Discussions

### Total soluble solids

A slight increase in the TSS of the roselle-fruit blends during 6 months of storage at both storage temperatures was observed. TSS for roselle-fruit blends ranged from 5.6 to 11.2 brix (28°C) and 5.6–12.0 brix (4°C) during the 6 months of storage (Table 1). Retention or minimum increase in TSS content of juice during storage is desirable for preservation of good juice quality (Bhardwaj and Pandey 2011)**.**

**Table 1:** Physicochemical and antioxidant properties of roselle-fruit blends stored 0–6 months at 28°C and 4°C.

		Mango			Papaya			Guava		
	Blends		28°C	4°C		28°C	4°C		28°C	4°C
Parameters		0	6	6	0	6	6	0	6	6
TSS	20R	10.6	11.0	12.0	6.9	6.0	8.8	5.6	5.8	6.4
	40R	9.9	11.2	11.2	7.6	6.3	8.3	5.9	6.2	6.8
	60R	7.5	7.4	10.2	7.8	7.7	8.3	6.3	6.9	7.7
	80R	6.9	7.6	7.5	6.7	8.4	7.3	6.7	7.5	6.6
pH	20R	2.76	2.70	3.03	3.32	4.37	3.38	3.13	3.17	3.25
	40R	2.65	2.57	2.77	2.94	2.93	2.92	2.53	2.58	2.64
	60R	2.40	2.43	2.56	2.69	2.65	2.64	2.83	2.60	2.59
	80R	2.34	2.42	1.90	2.54	2.50	2.54	2.41	2.47	2.40
TA	20R	2.92	1.44	1.44	1.36	2.40	1.36	1.92	1.24	1.92
	40R	3.12	1.44	1.32	2.92	1.28	1.28	1.36	2.40	1.36
	60R	2.34	2.92	2.40	1.60	1.44	1.60	2.40	1.96	1.96
	80R	1.92	2.40	1.40	2.00	1.68	1.92	1.68	2.88	1.68
RS	20R	3.48	6.36	7.35	2.95	2.95	7.00	3.32	6.97	7.01
	40R	4.51	5.22	8.46	3.45	7.70	7.71	3.88	8.16	7.92
	60R	5.06	7.87	8.93	4.87	8.19	8.87	4.10	7.96	8.58
	80R	5.55	9.92	9.32	5.18	9.24	9.24	4.35	9.19	8.98

80R, 80% roselle; 60R, 60% roselle; 40R, 40% roselle; 20R, 20% roselle; TSS, total soluble solids; TA, titratable acidity; RS, reducing sugars.

### pH

The roselle-fruit juice blends ranged from 2.34 to 4.37 (28°C) and 2.34–3.38 (4°C) during the 6 months of storage (Table 1). An increase in pH was observed at 28°C and 4°C in some roselle-fruit blends. The increased pH was due to the decrease in acidity of the juices. Fruit juices have a low pH because they are comparatively rich in organic acids (Tasnim et al. 2010). Kumar et al. 2012 also observed a significant increase in pH over a period of 120 days of storage at ambient temperature of guava blended with aloe vera and roselle juice nectars.

### Titratable acidity

TA for roselle-fruit juice blends ranged from 3.12 to 1.28 (28°C) and 3.12–1.24 (4°C) during the 6 months of storage (Table 1). The TA for some of the roselle-fruit juice blends was found to decrease significantly (*P* < 0.05) at 28°C as well as at 4°C (Tables 3 and 4). Decreased acidity might be due to acidic hydrolysis of polysaccharides where acid is utilized for converting non-RS into RS (Bhardwaj and Pandey 2011).

### Reducing sugars

Sugars are one of the most important constituents of fruit products, essential for and also act as a natural food preservative (Bhardwaj and Pandey 2011). The RS value for roselle-fruit ranged from 2.95 to 9.92 mg/100 g (28°C) and 2.95–9.32 mg/100 g (4°C) during the 6 months of storage. The results show a significant increase (*P* < 0.05) in RS with increasing storage period. The sugar content of fruit juices usually increases with increased storage period. The increase is probably due to the hydrolysis of polysaccharides like starch, cellulose, pectin, etc. and conversion into simple sugars (glucose, fructose). Kausar et al. (2012) reported increased RS with increased storage time of a cucumber–melon functional drink and 70% increased RS during the 6 months of storage of bottled gourd–basil leave juice (Majumdar et al. 2011).

### Effects of storage temperature and time on color

Visually, no color change was observed in all of the roselle-fruit blends during the 6 months of storage at 4°C. However, minimal loss in visual color was observed in all roselle-fruit blends stored for 4–6 months at 28°C. The results are similar to the findings of Saeed and Ahmed (1977), who did not observe any visual color change in carbonated beverages prepared from roselle calyces during 3 months of storage at ambient temperature.

Lightness values (L*) of the roselle-fruit blends ranged from 19.6 to 13.8 (28°C) and 19.6–14.2 (4°C) for 6 months of storage while redness values (a*) of the roselle-fruit blends ranged from 20.0 to 13.0 (28°C) and 20.0–13.5 (4°C) respectively after 6 months of storage (Table 2). Anthocyanins are responsible for the red color in roselle-mango juice blends and color of anthocyanin is pH dependent (the red flavylium is stable at low pH) as the pH changes were substantial hence the color changes of the roselle-fruit blends. Yellowness (b*) values of the roselle-fruit blends ranged from 8.5 to 2.7 (28°C) and 8.5–3.7 (refrigerated) during the 6 months of storage.

**Table 2:** Color parameters (lightness [L^*^], redness [a^*^] and yellowness [b^*^] of roselle-fruit blends stored 0–6 months at 28°C and 4°C.

Parameters	L^*^			a^*^			b^*^		
Storage temperature		28°C	4°C		28°C	4°C		28°C	4°C
Storage time	0	6	6	0	6	6	0	6	6
Mango									
20R	18.6	14.7	16.6	18.3	16.2	17.2	19.6	18.1	19.0
40R	17.6	15.2	16.1	17.8	15.8	17.1	16.3	14.7	15.6
60R	16.1	14.8	15.4	16.6	14.8	15.6	15.8	13.8	14.8
80R	14.7	13.8	14.5	15.8	13.8	15.1	15.1	14.2	14.2
Papaya									
20R	16.4	14.8	14.8	15.4	13.0	14.6	16.5	14.7	14.7
40R	17.6	16.1	14.3	16.2	13.5	13.5	17.2	16.2	16.2
60R	19.1	14.7	14.7	18.8	17.3	17.3	17.6	15.8	15.8
80R	20.0	17.9	17.4	19.1	17.5	19.1	19.5	17.4	17.4
Guava									
20R	8.5	6.8	7.4	7.3	5.4	6.7	7.3-	5.4	6.9
40R	7.7	5.8	5.3	6.4	5.4	4.9	6.4	5.4	5.4
60R	5.6	4.3	4.3	6.0	4.0	4.9	5.3	3.2	4.5
80R	4.6	3.9	3.9	4.9	2.7	4.3	4.5	2.7	3.7

80R, 80% roselle; 60R, 60% roselle; 40R, 40% roselle; 20R, 20% roselle.

### Effect of time and temperature of storage on vitamin C content

**Figure 1:**
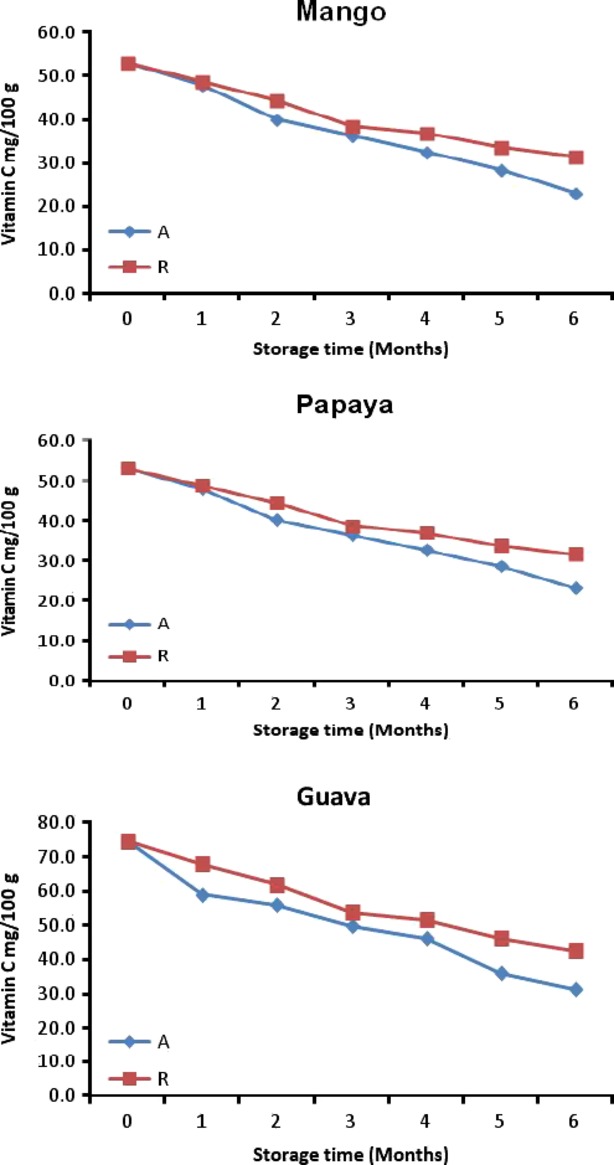
Vitamin C content for roselle-fruit blends (40R) stored for six months at ambient (A) and refrigerated (R).

The vitamin C contents for roselle-mango, roselle-papaya, roselle-guava juice (40R) blends were 54.4, 53.0, 74.7 mg/100 g FW initially and changed to 24.5, 23.2, 31.3 mg/100 g FW (28°C) and 31.5, 31.5 42.6 mg/100 g FW (4°C) during the 6 months of storage (Fig. 1). Vitamin C content of all roselle-fruit blends decreased during storage with the advancement of storage period, which was probably due to the fact that vitamin C being sensitive to oxygen, light and heat are easily oxidized in the presence of oxygen by both enzymatic and non-enzymatic catalysts (Ziena 2000). A decrease in vitamin C was observed in guava blended with aloe vera and roselle during 120 days of storage at ambient temperature (Kumar et al. 2012).

### Effect of time and temperature of storage on TMA

**Figure 2:**
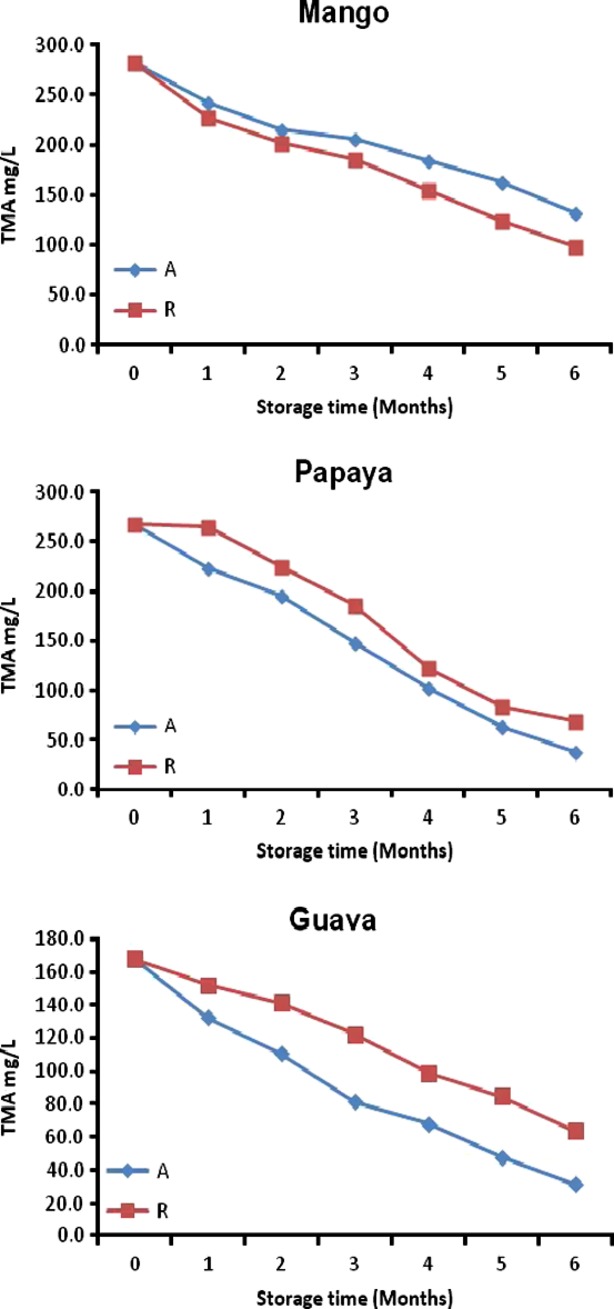
Total monomeric anthocyanin content for roselle-fruit blends (40R) stored for six months at ambient (A) and refrigerated (R).

**Figure 3:**
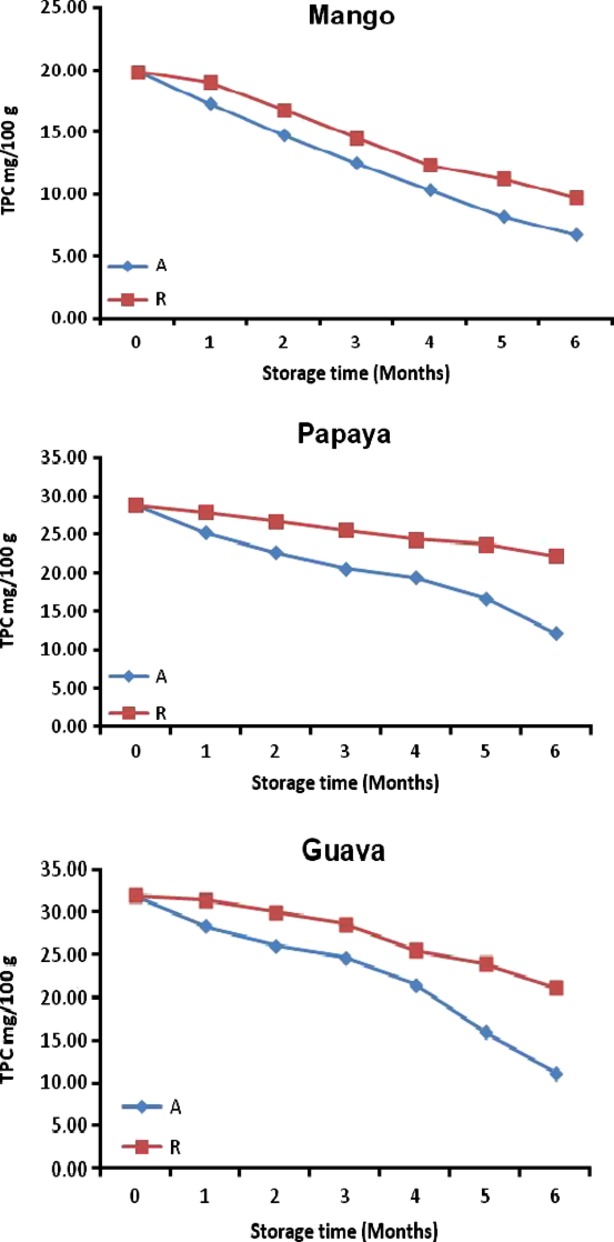
Phenolic content for roselle-fruit blends (40R) stored for six months at ambient (A) and refrigerated (R).

The TMA for roselle-fruit juice blends (40R) is shown in Figure 2. TMA for roselle-mango, roselle-papaya, and roselle-guava juice (40R) blends was 282.6, 268.6, and 167.8 mg/100 g FW initially and changed to 97.8, 37.4, and 31.3 mg/100 g FW (28°C) and 131.4, 68.0, and 63.7 mg/100 g FW (4°C) after 6 months of storage (Fig. 3). The losses in TMA for roselle-fruit juices were higher at 28°C than 4°C. The TMA for roselle-fruit juice blends was found to be decreased during storage but this decrease was statistically significant (*P* < 0.05) at 28°C as well as at 4°C (Tables 3 and 4). The presence of ascorbic acid and higher pH of the prepared roselle-fruit juice blends could have accelerated anthocyanin degradation. It is also known that interaction of ascorbic acid with anthocyanins may result in the degradation of both compounds through a condensation reaction (Choi et al. 2002; González-Molina et al. 2009). From the results roselle-guava blends with higher vitamin C content had greater loss of anthocyanin than roselle-mango and roselle-papaya blends.

**Table 3:** Influence of treatment effects on physicochemical and bioactive properties of roselle-fruit juice blends (40R).

Parameters	TSS	pH	TA	RS	Vit C	FRAP	TMA	TPC	L^*^	a^*^	b^*^
Fruit	<0.001	<0.001	<0.001	<0.001	<0.001	<0.001	<0.001	<0.001	<0.001	<0.001	<0.001
Temp	ns	<0.001	<0.001	<0.001	<0.001	<0.001	<0.001	<0.001	<0.001	<0.001	<0.001
Time	<0.001	<0.001	<0.001	<0.001	<0.001	<0.001	<0.001	<0.001	<0.001	<0.001	<0.001
Fruit × Temp	<0.001	<0.001	ns	<0.001	<0.001	<0.001	<0.001	<0.001	0.031	<0.001	<0.001
Fruit × Time	<0.001	<0.001	<0.001	<0.001	<0.001	<0.001	<0.001	<0.001	0.008	<0.001	<0.001
Temp × Time	<0.001	<0.001	<0.001	<0.001	<0.001	<0.001	<0.001	<0.001	<0.001	0.022	0.004

TSS, total soluble solids; TA, titratable acidity; RS, reducing sugars; Vit C, vitamin C; FRAP, ferric reducing ability of plasma; TMA, total monomeric anthocyanins; TPC, total phenolic content; ns, not significant.

**Table 4:** Main effects of fruit juice, storage temperature and time on the physicochemical and bioactive properties of roselle-fruit juice blends during storage.

Parameters	TSS	pH	TA	RS	Vit C	FRAP	TMA	TPC	L^*^	a^*^	b^*^
Fruit juice											
Mango	9.3a	2.6a	1.7a	6.4a	39.6b	1.2b	105.0b	25.2a	17.0a	16.9a	6.8a
Papaya	9.1b	2.6a	1.4b	6.0b	39.3c	1.2b	175.8a	19.2b	16.5b	15.2c	5.8c
Guava	6.2c	2.5b	1.4b	5.6c	53.7b	2.5a	177.8a	17.8c	15.7c	16.7b	6.2b
Storage temperature (°C)											
28	8.2a	2.5b	1.5b	5.6b	41.7b	1.6b	137.7b	16.8b	16.2b	16.1b	6.2b
4	8.2b	2.6a	1.6a	6.3a	46.7a	1.7a	168.1a	24.7a	16.6a	16.4a	6.4a
Storage time (months)											
0	7.7d	2.7a	1.5b	4.5e	60.7a	1.8a	236.5a	26.9a	17.2a	17.2a	6.8a
1	7.7d	2.7a	1.5b	4.6e	53.4b	1.8a	207.2b	24.9b	16.8b	16.9a	6.7a
2	8.2c	2.4c	1.4bc	4.6e	48.0c	1.8a	181.4c	22.9c	16.4c	16.6b	6.7a
3	8.2c	2.3d	1.3c	5.3d	42.9d	1.7b	154.6d	21.1d	16.2c	16.2bc	6.5b
4	8.4b	2.5b	1.4bc	6.5c	39.5e	1.5c	121.7c	18.9e	16.2c	16.1c	6.1c
5	8.5b	2.4c	1.6b	7.1b	34.6f	1.4d	94.1f	16.6f	16.1c	15.5d	5.7d
6	8.7a	2.7a	1.9a	8.5a	30.4g	1.3e	71.5 g	13.9 g	15.7d	15.2d	5.3c

TSS, total soluble solids; TA, titratable acidity; RS, reducing sugars; Vit C, vitamin C; FRAP, ferric reducing ability of plasma; TMA, total monomeric anthocyanins; TPC, total phenolic content; L^*^, lightness; a^*^, redness; b^*^, yellowness. Means separated in columns by main effects of Tukey's test. Numbers followed by the same letter are not significantly different (*P* < 0.05).

### Effect of time and temperature of storage on the total phenolic content

TPC for roselle-mango, roselle-papaya, roselle-guava juice (40R) blends were 19.8, 28.8, and 32.0 GAE mg/100 g FW initially and changed to 6.7, 12.2, 11.1 GAE mg/100 g FW (28°C) and 9.71, 22.3, 21.2 GAE mg/100 g FW (4°C) at 6 months of storage (Fig. 3). The data reveal that the TPC decreased during storage and significantly (*P* < 0.05) more decrease was found at 28°C than at 4°C, irrespective of storage intervals (Tables 3 and 4).

During storage, some monomeric anthocyanins might have been transformed into polymeric compounds (Iversen 1999; Ochoa et al. 1999). This might be the reason for less reduction of TPC and high losses in TMA in the blends.

### Antioxidant activity

**Figure 4:**
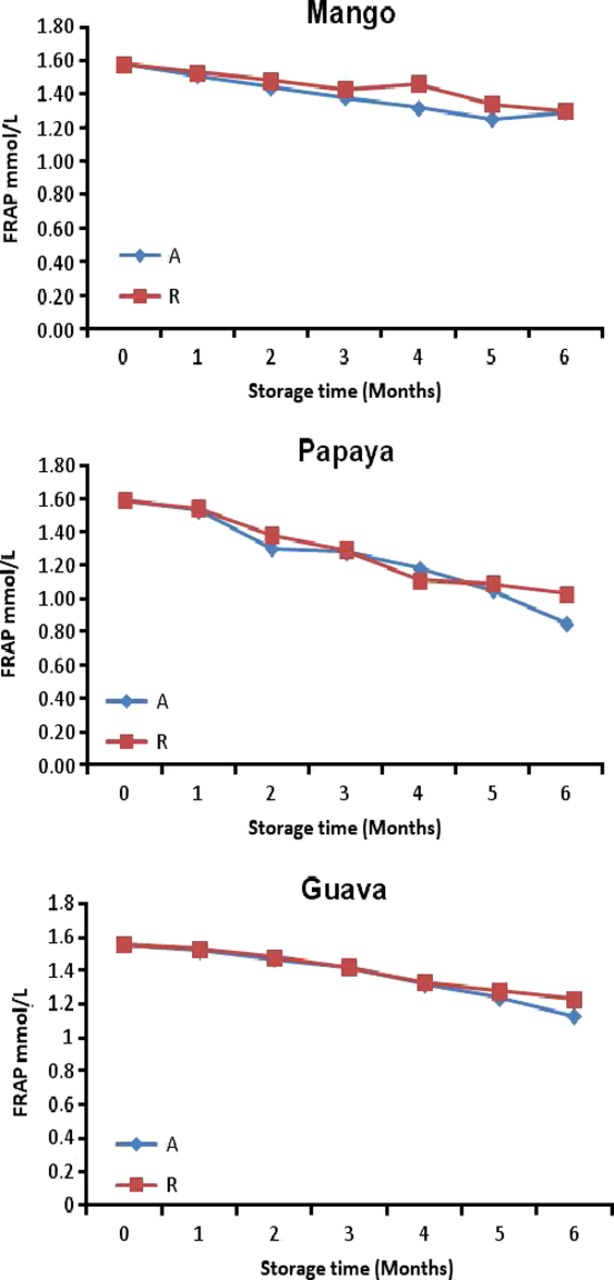
Antioxidant activity (FRAP) for roselle-fruit blends (40R) stored for six months at ambient (A) and refrigerated (R).

FRAP values for roselle-mango blends ranged from 1.8 to 0.76 mmol/100 g (28°C and 4°C) during the 6 months of storage (Fig. 4). The antioxidant capacity of fruits and vegetables, which benefits human health, is highly correlated with their anthocyanin and TPC (Fang et al. 2006). The results showed that antioxidant activity levels did not decrease substantially.

Despite marked losses of monomeric anthocyanins in the roselle-fruit juice blends, FRAP values were not higher during storage, suggesting the possibility of formation of polymeric compounds from monomeric anthocyanins during storage which were able to compensate the loss of antioxidant capacity due to decreased monomeric anthocyanins (Brownmiller et al. 2008).

### PCA of roselle-fruit blends

**Figure 5:**
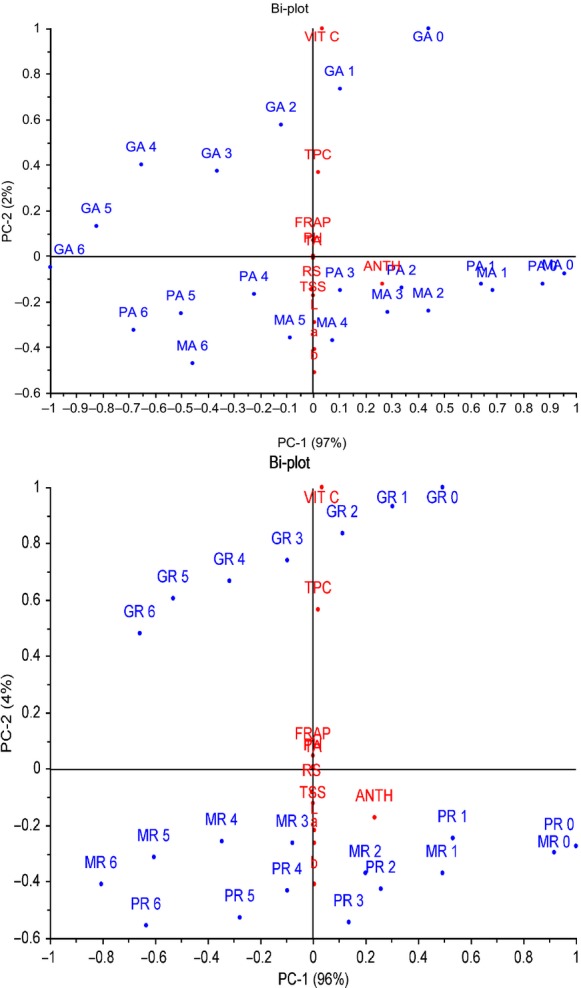
(A, B) Bi-plot (PCA) showing the effects of storage time and temperature on the roselle-fruit blends at ambient and refrigerated temperature.

A PCA was applied to characterize the different roselle-fruit blends by their storage time and storage temperature (Fig. 5A and B). The two principal components were able to explain all total variation. The principal component 1 (PC1) and PC2 divided the roselle-fruit juice blends into two clusters depending on the type of fruit mixed with roselle extract. PC2 was used to explain roselle-guava blends and was characterized by high levels of vitamin C, TPC, FRAP, and pH, while PC1 explained roselle-mango and roselle-papaya juice blends with high level of TMA, total soluble solids, RS, lightness (L*), redness (a*), and yellowness (b*).

The roselle-guava juice blends stored at ambient temperature formed a cluster with blends stored at 0 and 1 months on the positive side of PC2 and blends stored from 2 to 6 months on the negative side of PC. The roselle-papaya and roselle-mango juice blends form the second cluster with blends stored at 0–3 months (roselle-papaya) and 0–4 months (roselle-mango) on the positive side of PC1 and blends stored at 4–6 months (roselle-papaya) and 5–6 months (roselle-mango) on the negative side of the PC (Fig. 5A).

The roselle-guava juice blends stored at refrigerated temperature formed a cluster with blends stored at 0–2 months on the positive side of PC2 and blends stored from 3 to 6 months on the negative side of PC. The roselle-papaya and roselle-mango juice blends form the second cluster with blends stored at 0–3 months (roselle-papaya) and 0–2 (roselle-mango) on the positive side of PC1 and blends stored at 4–6 months (roselle-papaya) and 3–6 months (roselle-mango) on the negative side of the PC (Fig. 5B). Regardless of the storage time, TMA, TPC, and vitamin C were mostly affected during storage of roselle-fruit juice blends stored for 6 months. This shows that the storage temperature had a clear effect on the loss of TMA.

## Conclusions

The roselle-fruit blends have high content of vitamin C, anthocyanin, and total phenol. However, these compounds were lost during 6 months of storage at 28°C and 4°C. The loss of vitamin C and anthocyanin was more pronounced at 28°C; therefore, storage at 4°C should be encouraged when the products need to be stored for long time.
